# Non-Diabetic Glycosuria

**Published:** 1900-04-21

**Authors:** 


### NON-DIABETIC GLYCOSURIA.

Speaking at the London Polyclinic on the subject of Glycosuria, Dr. Robert Saundby recently pointed out how important it is in studying diabetes to study also the conditions under which non-diabetic glycosuria may occur. If on boiling a few drops of urine with about a drachm of Fehling's solution, previously ascertained to be not liable to spontaneous reduction, any change from blue to green takes place, or still more if a green or yellow precipitate occurs, the case requires further investigation. It is well known that other substances besides sugar may cause reduction of the copper, and the most satisfactory proof that this reduction is due to sugar is undoubtedly the production of carbonic acid gas by fermentation with yeast. This, however, takes time, and unless the yeast be washed is attended with certain fallacies; and for some years back Dr. Saundby has followed the plan, recommended by the late Sir William Roberts, of filtering a few drachms of the urine seven or eight times through animal charcoal. This only takes about ten minutes, and is found very satisfactory, as it removes from the urine all other reducing substances except sugar. Dr. Saundby refers to a few cases of alimentary or physiological glycosuria, but states that with these exceptions all the patients in whom he had found sugar had been ill. They may not have had diabetes, but they were ill. By far the largest number of them were suffering from disorders of the alimentary system; chronic gastritis, alcoholism, chronic hepatitis, biliary colic. The next most numerous group was the diathetic; gout, muscular rheumatism, uric acid, and oxalate calculi. Then came diseases of the nervous system, including neurasthenia, then infective processes, injuries, senile decay, cancer, and finally lactation. Dr. Saundby, however, especially insists on alcoholism as a cause of non-diabetic glycosuria, of which he relates several interesting examples; and he points out that alcohol was probably at the root of some of the cases which had been classed either as chronic hepatitis and chronic gastritis. In certain cases, however, there can be no doubt that hepatic glycosuria may occur independently of alcoholic excess, as for example, in malarial hepatitis and in hepatic abscess, and the same may be said in regard to gastric glycosuria. It is probable, as Dr. Saundby says, that all those cases included under alcohol, liver diseases, and gastritis have some connecting link, which, could we discover it, would throw light upon the true etiology of glycosuria. This factor is probably some derangement of one of the important organs concerned in digestion; and, from the well-known relation of the liver to sugar production, it would certainly seem most reasonable to regard this organ as the one most likely to be at fault, the probability being that in these conditions there are sudden discharges of sugar from the liver into the blood in quantities greater than can be used up in the body. Cases of non-diabetic glycosuria do not require the strict diet commonly ordered in diabetes and in senile glycosuria, which may be regarded as part of a general breakdown, much care must be exercised in this regard; and, although in these old people there can be no harm in stopping sugar and sweet wines, one should hesitate in cutting them off bread or potatoes or any farinaceous food they may be in the habit of taking.

